# Supplementary material for: Use of Nanostructured Silica SBA-15 as an Oral Vaccine Adjuvant to Control Mycoplasma hyopneumoniae in Swine Production
Source: Int J Mol Sci. 2023 Apr 1;24(7):6591. doi: 10.3390/ijms24076591 (PMC10095401; doi:10.3390/ijms24076591)
Supplement: Supplementary file 1 [file ijms-24-06591-s001.zip › ijms-2170508-supplementary.pdf]

International Journal of
Molecular Sciences

MDPI

Table S1. Means ± standard error of rectal temperature measurements recorded for groups 1 to 4 over the study period.

	Rectal temperature (ºC)[1]				
Day	G1	G2	G3	G4	P value
D3	38.96 ± 0.1	39.17 ± 0.08	38.99 ± 0.08	38.84 ± 0.07	6.02 x 10-2
D4	39.01 ± 0.07	39.03 ± 0.05	39.06 ± 0.07[a]	38.8 ± 0.06[b]	1.53 x 10-2
D5	38.90 ± 0.09	39.03 ± 0.06	39.21 ± 0.11	38.95 ± 0.09	9.55 x 10-2
D10	39.28 ± 0.19	39.14 ± 0.1	38.99 ± 0.12	38.88 ± 0.15	2.44 x 10-1
D17	37.95 ± 0.11[b]	38.17 ± 0.1	38.34 ± 0.11	38.39 ± 0.12[a]	3.05 x 10-2
D24	37.39 ± 0.16[b]	38.31 ± 0.12[a]	38.11 ± 0.16[a]	37.93 ± 0.09[a]	1.27 x 10-4
D26	37.85 ± 0.07[b]	38.29 ± 0.12[a]	38.12 ± 0.1	38.33 ± 0.09[a]	7.30 x 10-3
D31	37.85 ± 0.13[b]	38.29 ± 0.21[a]	38.12 ± 0.16	38.33 ± 0.23[a]	9.40 x 10-1
D41	37.10 ± 0.15	37.32 ± 0.19	37.01 ± 0.11	37.41 ± 0.19	4.55 x 10-1
D51	38.32 ± 0.15	38.33 ± 0.14	38.35 ± 0.14	37.84 ± 0.23	1.23 x 10-1
D61	38.76 ± 0.13	38.37 ± 0.08	38.54 ± 0.15	38.64 ± 0.15	2.56 x 10-1
D71	38.29 ± 0.19	38.19 ± 0.16	37.98 ± 0.23	38.37 ± 0.16	4.56 x 10-1

[1]Means followed by different letters in the same row differ significantly by Tukey's parametric test ($p < 0.05$).

Table S2. P-values from multiple comparisons between rectal temperature measurements recorded over time

Comparation	G1	G2	G3	G4
D3-D0	1.00[1]	1.00	1.00	1.00
D5-D0	1.00	1.00	1.00	1.00
D10-D0	1.00	1.00	1.00	1.00
D17-D0	9.88 x 10 -3	4.19 x 10 -4	1.50 x 10 -1	4.27 x 10 -1
D24-D0	9.16 x 10 -6	1.53 x 10 -2	4.61 x 10 -3	1.60 x 10 -3
D26-D0	6.35 x 10 -3	1.01 x 10 -2	6.46 x 10 -3	1.68 x 10 -1
D31-D0	3.85 x 10 -4	1.75 x 10 -7	4.40 x 10 -6	5.16 x 10 -4
D41-D0	1.72 x 10 -7	6.10 x 10 -8	5.82 x 10 -10	4.88 x 10 -7
D51-D0	6.14 x 10 -1	1.08 x 10 -2	8.14 x 10 -2	5.80 x 10 -3
D61-D0	1.00	1.53 x 10 -2	6.86 x 10 -1	9.53 x 10 -1
D71-D0	4.47 x 10 -1	1.99 x 10 -3	6.46 x 10 -3	2.67 x 10 -1
D5-D3	1.00	1.00	1.00	1.00
D10-D3	1.00	1.00	1.00	1.00
D17-D3	2.52 x 10 -3	1.45 x 10 -3	1.23 x 10 -1	5.95 x 10 -1
D24-D3	1.08 x 10 -6	3.68 x 10 -2	3.29 x 10 -3	3.95 x 10 -3
D26-D3	1.38 x 10 -3	2.42 x 10 -2	4.61 x 10 -3	2.80 x 10 -1
D31-D3	6.02 x 10 -5	1.61 x 10 -6	2.32 x 10 -6	1.50 x 10 -3
D41-D3	5.75 x 10 -8	2.54 x 10 -7	3.01 x 10 -10	1.07 x 10 -5
D51-D3	3.59 x 10 -1	2.58 x 10 -2	6.48 x 10 -2	1.41 x 10 -2
D61-D3	9.99 x 10 -1	3.68 x 10 -2	6.31 x 10 -1	9.87 x 10 -1
D71-D3	2.27 x 10 -1	6.07 x 10 -3	4.61 x 10 -3	4.09 x 10 -1
D10-D5	9.98 x 10 -1	1.00	9.99 x 10 -1	1.00
D17-D5	2.31 x 10 -2	9.52 x 10 -4	2.23 x 10 -2	3.27 x 10 -1

Continues on the next page

D24-D5	3.55×10^{-5}	2.92×10^{-2}	2.57×10^{-4}	8.04×10^{-4}
D26-D5	1.46×10^{-2}	1.79×10^{-2}	4.03×10^{-4}	1.15×10^{-1}
D31-D5	1.19×10^{-3}	1.28×10^{-6}	1.76×10^{-7}	2.74×10^{-4}
D41-D5	1.08×10^{-6}	3.05×10^{-8}	3.17×10^{-12}	1.61×10^{-7}
D51-D5	7.69×10^{-1}	2.01×10^{-2}	9.96×10^{-3}	3.11×10^{-3}
D61-D5	1.00	2.92×10^{-2}	2.54×10^{-1}	9.08×10^{-1}
D71-D5	6.14×10^{-1}	4.39×10^{-3}	4.03×10^{-4}	1.90×10^{-1}
D17-D10	4.23×10^{-4}	5.58×10^{-4}	2.67×10^{-1}	6.49×10^{-1}
D24-D10	7.06×10^{-8}	1.79×10^{-2}	1.24×10^{-2}	5.40×10^{-3}
D26-D10	3.20×10^{-4}	1.19×10^{-2}	1.58×10^{-2}	3.27×10^{-1}
D31-D10	7.99×10^{-6}	2.57×10^{-6}	3.47×10^{-5}	2.02×10^{-3}
D41-D10	1.04×10^{-9}	4.13×10^{-8}	7.92×10^{-9}	4.92×10^{-6}
D51-D10	1.59×10^{-1}	1.29×10^{-2}	1.60×10^{-1}	1.88×10^{-2}
D61-D10	9.85×10^{-1}	1.79×10^{-2}	8.42×10^{-1}	9.93×10^{-1}
D71-D10	8.75×10^{-2}	2.91×10^{-3}	1.58×10^{-2}	4.64×10^{-1}
D24-D17	9.53×10^{-1}	9.99×10^{-1}	9.96×10^{-1}	8.00×10^{-1}
D26-D17	1.00	1.00	9.98×10^{-1}	1.00
D31-D17	1.00	9.85×10^{-1}	3.77×10^{-1}	6.32×10^{-1}
D41-D17	6.69×10^{-1}	8.40×10^{-1}	9.96×10^{-3}	5.14×10^{-2}
D51-D17	8.99×10^{-1}	1.00	1.00	9.40×10^{-1}
D61-D17	5.51×10^{-2}	9.99×10^{-1}	9.99×10^{-1}	9.99×10^{-1}
D71-D17	9.64×10^{-1}	1.00	9.98×10^{-1}	1.00
D26-D24	9.76×10^{-1}	1.00	1.00	9.68×10^{-1}
D31-D24	1.00	6.11×10^{-1}	9.69×10^{-1}	1.00
D41-D24	1.00	2.64×10^{-1}	2.40×10^{-1}	9.59×10^{-1}
D51-D24	8.75×10^{-2}	1.00	1.00	1.00
D61-D24	2.35×10^{-4}	1.00	7.38×10^{-1}	2.01×10^{-1}
D71-D24	1.59×10^{-1}	1.00	1.00	9.17×10^{-1}
D31-D26	1.00	7.00×10^{-1}	9.53×10^{-1}	8.98×10^{-1}
D41-D26	7.54×10^{-1}	3.40×10^{-1}	2.03×10^{-1}	1.79×10^{-1}
D51-D26	8.42×10^{-1}	1.00	1.00	9.97×10^{-1}
D61-D26	3.75×10^{-2}	1.00	7.86×10^{-1}	9.68×10^{-1}
D71-D26	9.33×10^{-1}	1.00	1.00	1.00
D41-D31	9.83×10^{-1}	1.00	9.80×10^{-1}	9.91×10^{-1}
D51-D31	4.10×10^{-1}	6.83×10^{-1}	5.39×10^{-1}	1.00
D61-D31	3.63×10^{-3}	6.11×10^{-1}	4.43×10^{-2}	1.07×10^{-1}
D71-D31	5.76×10^{-1}	9.07×10^{-1}	9.53×10^{-1}	8.00×10^{-1}
D51-D41	1.32×10^{-2}	3.24×10^{-1}	2.23×10^{-2}	8.40×10^{-1}
D61-D41	5.84×10^{-6}	2.64×10^{-1}	2.61×10^{-4}	1.58×10^{-3}
D71-D41	2.93×10^{-2}	6.11×10^{-1}	2.03×10^{-1}	1.07×10^{-1}
D61-D51	9.08×10^{-1}	1.00	9.95×10^{-1}	3.92×10^{-1}
D71-D51	1.00	1.00	1.00	9.85×10^{-1}
D71-D61	8.00×10^{-1}	1.00	7.86×10^{-1}	9.91×10^{-1}

[1] p-values for comparison between time-points by linear mixed effect model ($p < 0.05$).

Table S3. Medians ± standard error of median of S/P of IgG recorded for groups 1 to 4 over the study period.

S/P values for IgG anti - *M. hyopneumoniae*[1]					
**Day**	**G1**	**G2**	**G3**	**G4**	**P value**
D3	1.69 ± 0.03[b]	0.6 ± 0.06[d]	1.85 ± 0.04[a]	1.02 ± 0.09[c]	1.52×10^{-10}
D10	1.39 ± 0.03[a]	0.39 ± 0.06[b]	1.54 ± 0.03[a]	0.78 ± 0.02[b]	5.44×10^{-11}
D17	1.61 ± 0.13[a]	0.4 ± 0.07[b]	1.99 ± 0.06[a]	0.79 ± 0.14[b]	3.77×10^{-9}
D24	0.94 ± 0.06[a]	0.1 ± 0.05[b]	1.58 ± 0.09[a]	0.41 ± 0.03[b]	7.45×10^{-11}
D31	1.29 ± 0.08[b]	0.17 ± 0.09[c]	2.06 ± 0.09[a]	0.65 ± 0.04[c]	7.30×10^{-11}
D41	0.76 ± 0.06[b]	-0.01 ± 0.03[d]	1.14 ± 0.06[a]	0.31 ± 0.02[c]	1.31×10^{-10}
D51	0.52 ± 0.05[b]	0.19 ± 0.05[c]	0.86 ± 0.05[a]	0.23 ± 0.06[bc]	2.98×10^{-7}
D61	0.43 ± 0.03	0.31 ± 0.09[b]	0.61 ± 0.05[a]	0.22 ± 0.07[b]	1.12×10^{-2}
D71	0.39 ± 0.07	0.48 ± 0.08	0.58 ± 0.06	0.38 ± 0.09	2.61×10^{-1}

[1] Medians followed by different letters on the same row differ significantly by Kruskal-Wallis test ($p < 0.05$).

Table S4. P-values from multiple comparisons between anti - *M. hyopneumoniae* IgG S/P measurements recorded over time

Comparisons	G1	G2	G3	G4
D71-D3	2.70×10^{-9}	8.98×10^{-1}	1.55×10^{-6}	1.66×10^{-3}
D71-D10	1.36×10^{-4}	9.99×10^{-1}	1.50×10^{-3}	1.16×10^{-1}
D71-D17	2.24×10^{-7}	1.00	3.00×10^{-10}	4.96×10^{-1}
D71-D24	4.53×10^{-2}	2.77×10^{-3}	5.85×10^{-3}	1.00
D71-D31	2.71×10^{-4}	1.60×10^{-1}	3.00×10^{-10}	8.71×10^{-1}
D71-D41	7.26×10^{-1}	2.25×10^{-6}	3.22×10^{-1}	8.98×10^{-1}
D71-D51	1.00	5.56×10^{-2}	9.69×10^{-1}	9.79×10^{-1}
D71-D61	1.00	9.69×10^{-1}	1.00	9.69×10^{-1}
D61-D3	2.70×10^{-9}	2.16×10^{-1}	3.99×10^{-7}	1.05×10^{-5}
D61-D10	1.36×10^{-4}	1.00	$\times 10^{-3}$	2.79×10^{-3}
D61-D17	2.24×10^{-7}	9.91×10^{-1}	4.49×10^{-10}	3.72×10^{-2}
D61-D24	4.53×10^{-2}	1.15×10^{-1}	4.37×10^{-3}	9.91×10^{-1}
D61-D31	2.71×10^{-4}	8.40×10^{-1}	4.49×10^{-10}	1.86×10^{-1}
D61-D41	7.26×10^{-1}	8.59×10^{-4}	2.84×10^{-1}	1.00
D61-D51	1.00	5.90×10^{-1}	9.57×10^{-1}	1.00
D51-D3	1.51×10^{-7}	2.46×10^{-4}	2.12×10^{-4}	1.05×10^{-5}
D51-D10	9.04×10^{-4}	2.83×10^{-1}	8.11×10^{-2}	3.60×10^{-3}
D51-D17	4.72×10^{-6}	9.71×10^{-2}	1.05×10^{-6}	4.55×10^{-2}
D51-D24	1.36×10^{-1}	9.95×10^{-1}	1.86×10^{-1}	9.95×10^{-1}
D51-D31	1.51×10^{-3}	1.00	1.05×10^{-6}	2.16×10^{-1}
D51-D41	9.21×10^{-1}	3.62×10^{-1}	9.57×10^{-1}	1.00
D41-D3	8.11×10^{-5}	1.45×10^{-10}	3.04×10^{-2}	1.00×10^{-6}
D41-D10	9.64×10^{-2}	9.60×10^{-5}	7.26×10^{-1}	8.76×10^{-4}
D41-D17	2.54×10^{-3}	8.25×10^{-6}	7.58×10^{-4}	1.52×10^{-2}
D41-D24	8.98×10^{-1}	8.98×10^{-1}	8.98×10^{-1}	9.57×10^{-1}
D41-D31	1.36×10^{-1}	1.60×10^{-1}	7.58×10^{-4}	9.73×10^{-2}

Continues on the next page

D31-D3	5.91×10^{-1}	1.57×10^{-3}	9.86×10^{-1}	1.86×10^{-1}
D31-D10	1.00	5.43×10^{-1}	2.16×10^{-1}	9.21×10^{-1}
D31-D17	9.57×10^{-1}	2.49×10^{-1}	1.00	1.00 -
D31-D24	9.21×10^{-1}	9.41×10^{-1}	9.66×10^{-2}	7.67×10^{-1}
D24-D3	3.04×10^{-2}	2.25×10^{-6}	6.37×10^{-1}	7.78×10^{-4}
D24-D10	8.71×10^{-1}	3.00×10^{-2}	1.00	6.75×10^{-2}
D24-D17	2.17×10^{-1}	5.78×10^{-3}	9.66×10^{-2}	3.63×10^{-1}
D17-D3	9.98×10^{-1}	8.05×10^{-1}	9.86×10^{-1}	5.43×10^{-1}
D17-D10	9.79×10^{-1}	1.00	2.16×10^{-1}	9.98×10^{-1}
D10-D3	6.82×10^{-1}	4.96×10^{-1}	8.40×10^{-1}	9.41×10^{-1}

[1] p-values for comparison between time-points by Friedman test ($p < 0.05$).

Table S5. Medians ± standard error of median of S/P for IgA recorded for groups 1 to 4 over the study period.

S/P values for IgA[1]					
**Day**	**G1**	**G2**	**G3**	**G4**	**P value**
D3	0.32 ± 0.1	0.17 ± 0.05	0.3 ± 0.06	0.34 ± 0.06	3.09×10^{-1}
D10	0.08 ± 0.03[a]	-0.03 ± 0.09[b]	0.12 ± 0.03[a]	-0.02 ± 0.03[b]	2.19×10^{-3}
D17	0 ± 0.02[b]	-0.02 ± 0.02[b]	0.03 ± 0.09	0.08 ± 0.08[a]	1.51×10^{-2}
D24	0.17 ± 0.13[a]	0 ± 0.05	-0.02 ± 0.05	-0.09 ± 0.04[b]	3.50×10^{-2}
D31	0.28 ± 0.05[b]	0.44 ± 0.06	0.53 ± 0.07[a]	0.29 ± 0.07[b]	3.31×10^{-3}
D41	0.28 ± 0.12	0.28 ± 0.14	0.4 ± 0.09	0.37 ± 0.13	6.38×10^{-1}
D51	0.61 ± 0.06	0.62 ± 0.07	0.54 ± 0.08	0.44 ± 0.15	5.11×10^{-1}
D61	0.91 ± 0.12[a]	0.99 ± 0.12[a]	0.59 ± 0.12[b]	0.97 ± 0.24	2.12×10^{-2}
D71	1.22 ± 0.09	1.08 ± 0.15	0.81 ± 0.1[b]	1.81 ± 0.23[a]	4.39×10^{-2}

[1] Medians followed by different letters on the same row differ significantly by Kruskal-Wallis test ($p < 0.05$).

Table S6. P-values from multiple comparisons between anti - *M. hyopneumoniae* IgA S/P measurements recorded over time

Comparisons	G1	G2	G3	G4
D71-D3	1.55×10^{-3}	4.44×10^{-4}	4.42×10^{-3}	8.14×10^{-2}
D71-D10	6.69×10^{-7}	1.83×10^{-6}	2.60×10^{-5}	1.48×10^{-9}
D71-D17	9.37×10^{-11}	2.12×10^{-6}	6.53×10^{-6}	6.32×10^{-6}
D71-D24	5.97×10^{-5}	1.27×10^{-5}	9.10×10^{-8}	1.08×10^{-10}
D71-D31	2.67×10^{-3}	1.86×10^{-1}	8.98×10^{-1}	4.60×10^{-3}
D71-D41	3.65×10^{-3}	1.86×10^{-1}	3.62×10^{-1}	2.16×10^{-1}
D71-D51	3.63×10^{-1}	9.79×10^{-1}	9.21×10^{-1}	5.90×10^{-1}
D71-D61	1.00	1.00	9.69×10^{-1}	9.79×10^{-1}
D61-D3	1.22×10^{-2}	2.13×10^{-4}	1.60×10^{-1}	6.37×10^{-1}
D61-D10	1.27×10^{-5}	2.12×10^{-6}	3.46×10^{-3}	2.83×10^{-6}
D61-D17	4.00×10^{-8}	1.64×10^{-7}	2.16×10^{-3}	1.61×10^{-3}
D61-D24	6.72×10^{-4}	1.60×10^{-5}	4.63×10^{-6}	2.28×10^{-6}
D61-D31	1.97×10^{-2}	1.16×10^{-1}	1.00	1.36×10^{-1}

Continues on the next page

D61-D41	2.39×10^{-2}	1.16×10^{-1}	9.69×10^{-1}	8.71×10^{-1}
D61-D51	7.26×10^{-1}	9.41×10^{-1}	1.00	9.95×10^{-1}
D51-D3	6.82×10^{-1}	3.02×10^{-2}	2.48×10^{-1}	9.86×10^{-1}
D51-D10	2.95×10^{-2}	4.44×10^{-4}	7.41×10^{-3}	1.67×10^{-4}
D51-D17	1.82×10^{-4}	2.13×10^{-4}	4.42×10^{-3}	3.72×10^{-2}
D51-D24	2.16×10^{-1}	2.47×10^{-3}	2.60×10^{-5}	4.43×10^{-5}
D51-D31	7.67×10^{-1}	8.40×10^{-1}	1.00	6.37×10^{-1}
D51-D41	8.05×10^{-1}	8.40×10^{-1}	9.91×10^{-1}	1.00
D41-D3	1.00	7.26×10^{-1}	8.40×10^{-1}	1.00
D41-D10	7.67×10^{-1}	1.16×10^{-1}	1.36×10^{-1}	2.65×10^{-3}
D41-D17	8.06×10^{-2}	6.75×10^{-2}	9.66×10^{-2}	1.86×10^{-1}
D41-D24	9.91×10^{-1}	2.84×10^{-1}	1.47×10^{-3}	8.83×10^{-4}
D41-D31	1.00	1.00	9.95×10^{-1}	9.41×10^{-1}
D31-D3	1.00	7.26×10^{-1}	2.84×10^{-1}	9.95×10^{-1}
D31-D10	8.05×10^{-1}	1.16×10^{-1}	9.50×10^{-3}	1.60×10^{-1}
D31-D17	9.74×10^{-2}	6.75×10^{-2}	5.55×10^{-3}	9.22×10^{-1}
D31-D24	9.95×10^{-1}	2.84×10^{-1}	3.74×10^{-5}	8.14×10^{-2}
D24-D3	9.98×10^{-1}	9.99×10^{-1}	2.16×10^{-1}	4.60×10^{-3}
D24-D10	9.98×10^{-1}	1.00	9.21×10^{-1}	1.00
D24-D17	5.43×10^{-1}	1.00	9.57×10^{-1}	8.05×10^{-1}
D17-D3	1.36×10^{-1}	9.41×10^{-1}	9.21×10^{-1}	4.05×10^{-1}
D17-D10	9.41×10^{-1}	1.00	1.00	9.22×10^{-1}
D10-D3	8.71×10^{-1}	9.79×10^{-1}	9.57×10^{-1}	1.23×10^{-2}

[1] p-values for comparison between time-points by Friedman test ($p < 0.05$).
